# Demixing by a Nematic Mean Field: Coarse-Grained Simulations of Liquid Crystalline Polymers

**DOI:** 10.3390/polym9030088

**Published:** 2017-03-03

**Authors:** Abelardo Ramírez-Hernández, Su-Mi Hur, Julio C. Armas-Pérez, Monica Olvera de la Cruz, Juan J. de Pablo

**Affiliations:** 1 Materials Science Division, Argonne National Laboratory, 9700 South Cass Avenue, Argonne, IL 60439, USA; 2 Institute for Molecular Engineering, The University of Chicago, Chicago, IL 60637, USA; shur@chonnam.ac.kr (S.-M.H.); jc.armas@ugto.mx (J.C.A.-P.); 3 School of Polymer Science and Engineering, Chonnam National University, Gwangju 500-757, Korea; 4 División de Ciencias e Ingenierías, Campus León, Universidad de Guanajuato, Loma del Bosque 103, León, Guanajuato 37150, Mexico; 5 Department of Materials Science and Engineering, Northwestern University, Evanston, IL 60208, USA; m-olvera@northwestern.edu

**Keywords:** liquid crystalline polymers, coarse grained models, polymer blends

## Abstract

Liquid crystalline polymers exhibit a particular richness of behaviors that stems from their rigidity and their macromolecular nature. On the one hand, the orientational interaction between liquid-crystalline motifs promotes their alignment, thereby leading to the emergence of nematic phases. On the other hand, the large number of configurations associated with polymer chains favors formation of isotropic phases, with chain stiffness becoming the factor that tips the balance. In this work, a soft coarse-grained model is introduced to explore the interplay of chain stiffness, molecular weight and orientational coupling, and their role on the isotropic-nematic transition in homopolymer melts. We also study the structure of polymer mixtures composed of stiff and flexible polymeric molecules. We consider the effects of blend composition, persistence length, molecular weight and orientational coupling strength on the melt structure at the nano- and mesoscopic levels. Conditions are found where the systems separate into two phases, one isotropic and the other nematic. We confirm the existence of non-equilibrium states that exhibit sought-after percolating nematic domains, which are of interest for applications in organic photovoltaic and electronic devices.

## 1. Introduction

The self-assembly of soft matter systems provides a powerful tool to create complex hierarchical materials [1,2]. The structural organization at different scales, from atomistic to mesoscopic levels, is controlled by the different interactions between the component molecular units [3,4]. In the particular case of polymeric systems, such as block copolymers, those interactions are typically weak and isotropic, with the entropy playing an important role in molecular organization. Even with weak interactions, a plethora of domain structures, or morphologies, can be obtained by engineering their molecular architecture and composition [5]. The incorporation of molecular motifs governed by anisotropic interactions into the polymer backbone can give place to a richer array of phase behaviors [6,7]. Such anisotropic interactions can promote the long-range orientational organization of small molecules, and such orientational order can coexist with liquid-like characteristics for translational order, in nematic phases [8]. Thus, the incorporation of nematic structural units into polymer chains provides a rich platform for design of new materials with tunable optical, rheological and structural properties. The interplay of orientational interactions, phase segregation and entropy can give rise to new, complex hierarchical structures [9,10,11].

Liquid crystalline (LC) polymers have been used primarily to create materials that exhibit interesting mechanical properties [12]. However, in recent years, interest in these materials has increased due to their optical and transport properties [13,14,15,16,17]. In particular, LC polymers are common in organic photovoltaic applications and in flexible organic electronics [14]. In both cases, phase separated materials are required; for example, in the case of photovoltaic devices, one phase transports electrons and the other transports holes. It is also required that continuous pathways for transport permeate the entire sample in order to minimize recombination between electrons and holes [18]. More importantly, due to the very short lifetimes of excitons in organic materials, interfaces, where charge separation occurs, should be abundant [19]. Co-continuous morphologies represent a class of mesostructures that satisfy these conditions. Whereas thermodynamically stable morphologies would be preferred, non-equilibrium structures can also be very useful [20,21] if the associated relaxation times are sufficiently long. Thus, for example, some of the structures that appear during spinodal decomposition could be arrested by freezing the sample below the glass transition temperature, and used for developing photovoltaic devices.

Whereas significant work has been devoted to the study of phase separation in polymer mixtures, only a limited number of simulation efforts have focused on liquid crystalline polymers. On the theoretical side, several works have addressed the effect of chain stiffness and orientational coupling strength and the phase behavior of the corresponding mixtures [22,23,24,25,26,27,28,29]. The approach used in those reports consisted of approximating the exact free energy function by a Landau-Ginzburg expansion and, from that, a mapping to the phase diagram was carried out (see for example Ref. [23,25,29]). Such powerful theoretical tools provide considerable information about the behavior of liquid crystalline polymer mixtures. However, they entail approximations that are only valid in a certain regime, for example, large molecular weights or infinite chain stiffness. Recently, new studies have relied on self-consistent field theory (SCFT) [7,30] and single-chain in a mean field (SCMF) simulations to explore the physics of these systems [31]. However, most of those reports were focused on 2D systems [15,32,33] and, in the case of SCFT, fluctuations were neglected by the mean field approximation. In this work, we use a 3D particle-based coarse grained approach to study the effects of composition, persistence length, molecular weight and orientational coupling strength on the behavior of pure polymer systems and mixtures. Our results are summarized in the form of phase diagrams that could serve as a guide for experimental deployment of the systems considered here and, in future work, for formulation of polymeric blends for photovoltaic applications.

## 2. Model and Simulation Approach

In the interest of generality, we present the model in the context of an A-B polymer blend. The melt is composed by $n=n_{A}+n_{B}$ polymer chains in a volume *V* at temperature *T*. Macromolecules are modeled as discrete worm-like chains, and each chain consists of $N_{\gamma }$ polymer segments of length $b_{\gamma }$, with the index *γ* denoting one or the other type of polymer, γ=A,B. The conformation of a chain is described by the position of $N_{\gamma }+1$ nodes along the polymer backbone. The position of the *s*th node in the *i*th chain is denoted by $r_{i}\left(s\right)$. Polymer conformations are governed by a bending Hamiltonian which reads:

(1) $$\frac{H_{b}}{k_{B}T}=-\sum_{i=1}^{n}\sum_{s=1}^{N_{\gamma }}\kappa_{\gamma }\;{\hat{b}}_{i}\left(s+1\right)\cdot {\hat{b}}_{i}\left(s\right),$$

where ${\hat{b}}_{i}\left(s\right)=\left[r_{i}\left(s+1\right)-r_{i}\left(s\right)\right]/b_{\gamma }$ is the unit vector connecting nodes *s* and s+1, $k_{B}$ is the Boltzmann constant, and $\kappa_{\gamma }$ is the parameter that controls the chain stiffness of the corresponding polymer type, *γ*. The persistence length, $\ell_{p}$, and *κ*, are related by: bℓp=−ln[L(κ)], where L(x)=coth(x)−1x is the Langevin function [34]. To describe the intermolecular interactions we resort to a field-based approach [35]. The incompatibility between unlike segments is represented by a Flory-Huggins term, where *χ* parameterizes the strength of these interactions. A second term is included to constrain density fluctuations and assign a finite compressibility to the melt. This is achieved by using the Helfand’s quadratic approximation, with the strength of such interactions quantified by $\bar{\kappa }$ [36]. Finally, following [37,38], the orientational coupling is introduced by using a second-order term in the tensor order parameter Q(r), with the strength of this orientational interaction given by *μ*. Thus, the intermolecular interactions are given by the following functional:

(2) $$\begin{array}{ccc} \frac{H_{nb}}{k_{B}T} & = & \rho_{o}\int_{V}dr\left[\chi \phi_{A}\left(r\right)\phi_{B}\left(r\right)+\frac{\bar{\kappa }}{2}{\left[1-\phi_{A}\left(r\right)-\phi_{B}\left(r\right)\right]}^{2}-\frac{\mu }{3}Q\left(r\right):Q\left(r\right)\right], \end{array}$$

where $\phi_{\gamma }\left(r\right)$ is the local dimensionless density of segments of type *γ*, and $\rho_{o}$ is the segment number density. The microscopic definitions of these density and tensor fields are given in terms of polymer conformations by

(3) $$\phi_{\gamma }\left(r\right)=\sum_{i=1}^{n}\sum_{s=1}^{N_{\gamma }}\pi_{\gamma ,\gamma_{i(s)}}\delta \left({\bar{r}}_{i}\left(s\right)-r\right),$$

where $\gamma_{i(s)}$ is the type of segment *s* at chain *i*, and $\pi_{\gamma ,\gamma_{i(s)}}=1$ if $\gamma_{i(s)}=\gamma$, and equal to 0 otherwise. In this expression, ${\bar{r}}_{i}\left(s\right)$ denotes the center of mass of the corresponding segment, and it is given in terms of the node positions by: ${\bar{r}}_{i}\left(s\right)=\left[r_{i}\left(s+1\right)+r_{i}\left(s\right)\right]/2$.

(4) $$Q\left(r\right)=\rho_{o}^{-1}\sum_{i=1}^{n}\sum_{s=1}^{N_{\gamma }}\delta \left({\bar{r}}_{i}\left(s\right)-r\right)\left[\frac{3}{2}u_{i}\left(s\right)u_{i}\left(s\right)-\frac{I}{2}\right]$$

Here, the unit vector $u_{i}\left(s\right)$ describes the orientation of the corresponding polymer segment, and I is the unity tensor. In this work, we have used a simple bonded interaction (Equation (1)) and, for this case, $u_{i}\left(s\right)$ and ${\hat{b}}_{i}\left(s\right)$ represent the same object. We have used a different notation to anticipate the use of more complex intramolecular interactions.

The exploration of the self-assembled morphologies is performed by Monte Carlo simulations [39]. We use a particle-to-mesh technique where a grid is introduced, and local densities as well as local tensor fields are defined on each grid cell. The size of such cells are denoted by ΔL. The configurations are sampled according to the Metropolis criteria, $P_{\mathrm{acc}}=\min\left[1,\exp\left(-\Delta H/k_{B}T\right)\right]$, where ΔH is the energy difference between the original and a trial configuration. Note that it includes both intra- and inter-molecular contributions. The trial moves considered here include reptation-like displacements and local moves (end-rotation and flip moves) of the polymer segments. In the present report we focus on bulk systems; periodic boundary conditions are applied in all directions. We also note here that a similar approach was used in [37] to explore the case of a pure melt for a particular value of the chain stiffness parameter. In this work, we build on that work and explore a wider range of parameters as well as more complex molecular systems, including mixtures.

## 3. Results

### 3.1. Pure Systems

We begin by exploring the phase behavior of pure homopolymer melts as the orientational coupling strength, molecular weight and chain stiffness are varied. We choose χ=0, $\rho_{o}=1/b^{3}$, ΔL=2.5b and $\bar{\kappa }=6.0$. The latter value ensures that the system doesn’t collapse for the values of the orientational coupling, *μ*, studied in this work [37]. The unit length is chosen to be the polymer segment length, *b*. To characterize the global orientational order we compute the largest eigenvalue, *S*, of the total tensor parameter $Q_{\mathrm{tot}}=\int_{V}Q\left(r\right)\;dr$. Note that *S* is an uniaxial order parameter. We have performed Monte Carlo simulations on cubic simulation boxes of size $L_{x,y,z}=L$. To minimize finite-size effects we chose $L=5\ell_{c}$ (for melts with N= 4 and 8) and $L=3\ell_{c}$ (for melts with N= 16 and 24, where $\ell_{c}=Nb$ is the contour length associated to the specific molecular weight. All simulations were started from random initial configurations and evolved for 4 × $10^{6}$ MC steps.

**Figure 1:**
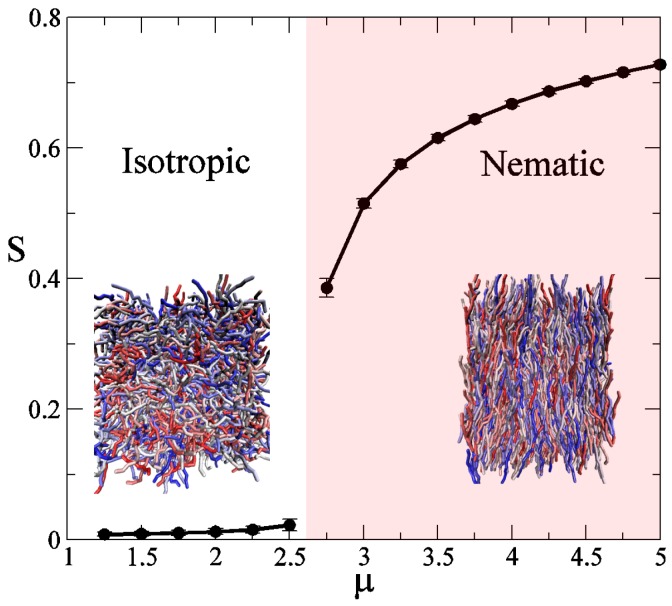
Global order parameter, S, as a function of the orientational coupling *μ*, obtained by Monte Carlo simulations (symbols). Polymer chains are composed of N=8 segments, and κ=3.5. Lines are only a guide to the eye. Insets are instantaneous polymer configurations in the isotropic and nematic phases, different chain colors are used to facilitate visualization.

Figure 1 illustrates the behavior of a liquid crystalline polymer melt with N=8 segments per chain. For this example, κ=3.5, which corresponds to a persistence length of approximately $\ell_{p}\approx 3b$. As can be seen in Figure 1, at small values of the orientational coupling, *μ*, the system displays an isotropic phase where chains are in a disordered state, with a global order parameter S≈0. However, there is a parameter value, $\mu_{t}\approx 2.7$, above which the global order parameter S≠0. The abrupt transition of this order parameter at $\mu =\mu_{t}$ is a signature of a first order phase transition, in this case between an isotropic and a nematic phase; the insets in Figure 1 show two instantaneous configurations of such phases. For clarity, we have unwrapped polymer positions from the simulation box. It should be noted that the isotropic-nematic transition does not entail a high degree of order (large S values), in agreement with experimental observations [40]. Note that in the first study our estimation of the orientational coupling at the transition, $\mu_{t}$, is not rigorous. Formally, more demanding free-energy calculations are needed to determine those values accurately as well as the order of the corresponding phase transition. Given that we use a finite set of equally spaced *μ* values, with spacing δμ, we approximate $\mu_{t}$ as the middle point between the largest value $\mu_{+}$ that gives $S(\mu_{+})\approx 0$, and the smallest value $\mu_{-}$ with $S(\mu_{-})\neq 0$, i.e., $\mu_{t}=\left(\mu_{-}+\mu_{+}\right)/2\pm \delta \mu /2$. Our simulations suggest a first order phase transition, and this agrees with the corresponding transition in small molecule liquid crystals, but more importantly, this also agrees with simulation results for semi-flexible polymers using a microscopic, Lennard-Jones-type model and mean-field predictions [41,42].

**Figure 2:**
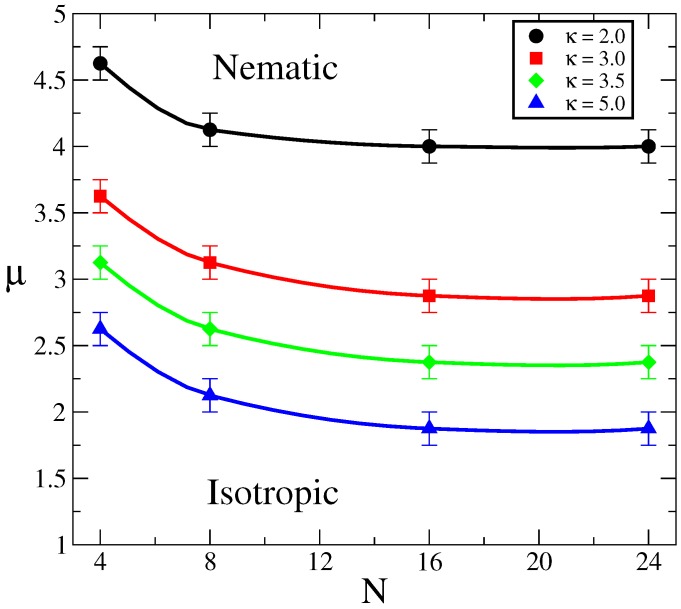
Phase diagram in the parameter space (*μ*, *N*) for different degrees of flexibility, parametrized by *κ*, obtained by Monte Carlo simulations. Symbols indicate the transition values, $\mu_{t}$, at which the isotropic-nematic transition occurs. Lines are only a guide to the eye. The associated persistence lengths, from small to large *κ* values, are: $\ell_{p}/b\approx 1.6,2.5,3.0$ and 4.5.

Next, we explore the effect of molecular weight, orientational coupling strength and chain stiffness on the phase behavior of liquid crystalline polymer melts. We summarize the results as a phase diagram on the parameter space (*μ*, *N*) for different stiffness, *κ*. The results are presented in Figure 2. As can be seen, chain stiffness has a strong effect on the location of the phase boundary between nematic and isotropic phases. As expected, more flexible chains (low *κ* values) require larger orientational coupling strength to induce nematic ordering. This is because conformational entropy is larger for such chains compared to rigid ones.

**Figure 3:**
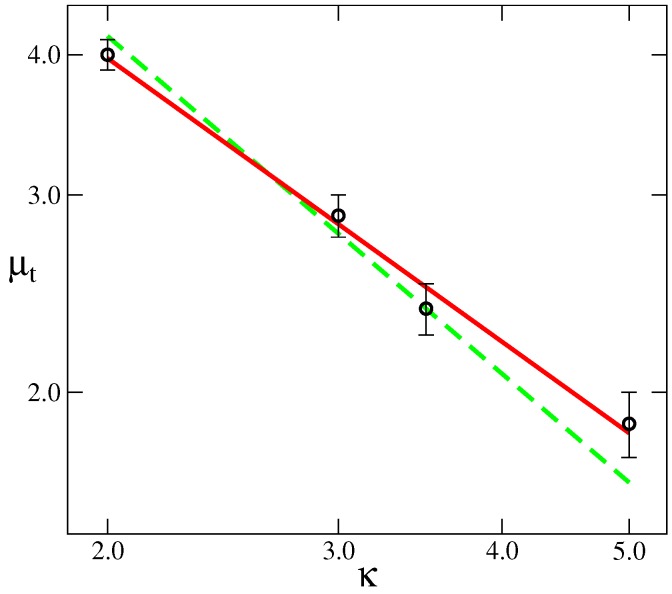
Relationship between the orientational coupling at the I-N transition, $\mu_{t}$, and the degree of flexibility, parametrized by *κ*, obtained by Monte Carlo simulations (symbols) at N=24. Green (dashed) line is the mean field prediction in the limit of very large N, $\mu_{t}∼\kappa^{-1}$. The red (continuous) line is a fit to a power law $\mu_{t}∼\kappa^{-\alpha }$, with exponent α≈0.84±0.05.

The polymerization index, *N*, also affects the transition $\mu_{t}$ values, although weakly. The larger *N*, the lower $\mu_{t}$ must be in order to induce orientational order. We should note here that Warner et al. [23] developed a mean field theory for nematic polymers and found that, in the limit of very large molecular weight, *N*, the orientational coupling at the isotropic-nematic transition, $\mu_{t}$, and the chain flexibility are related by $\mu_{t}∼\kappa^{-1}$. A simple argument to understand how this relationship arises can be written as follows: let’s consider the continuum limit of a worm-like chain in the presence of an “external” orientational potential μf[t(s)], where t(s) is the normalized tangent vector at the contour position *s*. The total Hamiltonian describing this chain is: HwlkBT=∫0Ndsκdt(s)ds2+μf[t(s)]. In the case of very large *κ* (≫1), the persistence length is given by $\ell_{p}=\kappa b$ (see Section 2 or Ref. [34]), thus it is possible to rescale the contour parameter *s* in terms of $\xi =sb/\ell_{p}$. Then, the energy of the chain is written as HwlkBT=∫0Nb/ℓpdξdtdξ2+μκf[t]; thus, in the limit of N→∞, the only parameter controlling the behavior is the product μκ. For a given value of this product, *a*, the free energy is well defined and μκ=a is satisfied. In order to compare such a prediction with our MC simulation results, using the data plotted in Figure 2, we can see that the cases with N=24 seem to have reached a constant value; we have used those values and plotted $\mu_{t}$ vs *κ*, in Figure 3. As can be seen, the simulation results deviate from the Warner et al. mean field predictions. The MC results follow a power law $\mu_{t}∼\kappa^{-\alpha }$, with α≈0.84; we ascribe such deviations in the scaling exponent to the effect of fluctuations that are included in our approach, but not present in the mean field approximation. Note, in particular, that the limits N→∞ and κ≫1, are critical to deduce that $\mu_{t}∼\kappa^{-1}$, and neither of these conditions are strictly satisfied in our calculations.

**Figure 4:**
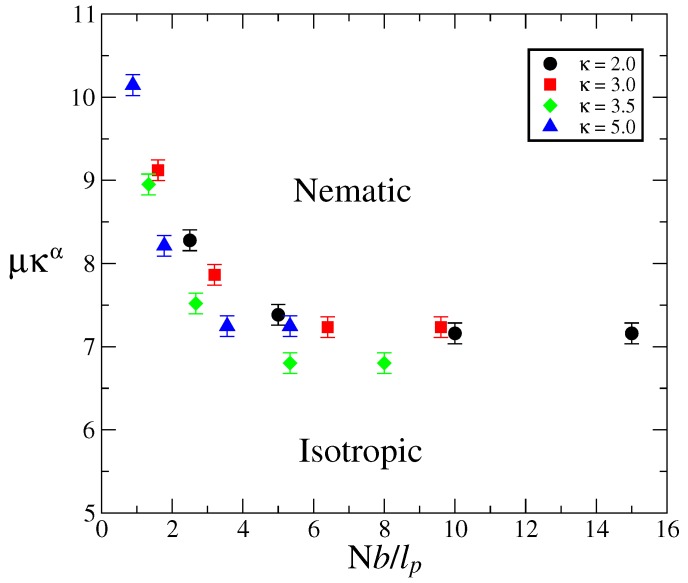
Phase diagrams in Figure 2 in terms of the renormalized parameters $\mu \kappa^{\alpha }$ and $Nb/\ell_{p}$.

Now, as can be seen in Figure 2, the curves for different chain stiffness exhibit similar shapes, so it is tempting to renormalize both the orientational coupling and polymerization index in search of a master curve. To that end, note that Figure 3 suggests that the important parameter, at large *N*, is the product $\mu \kappa^{\alpha }$, and on the other hand, what is important is the effective molecular length $Nb/\ell_{p}$, as opposed to the bare polymerization index. Therefore, we use these renormalized parameters to replot the phase diagrams in Figure 2. As can be seen in Figure 4, although the collapse is not perfect, all data points seem to accumulate around a common phase boundary.

A detailed comparison between mean field predictions regarding other properties is interesting but it is not main goal of the present work. We simply wish to highlight the fact that the simulation results agree with previous works and the approach is physically well defined. Thus, we proceed to use the model to exploring the behavior of more complex macromolecular systems.

### 3.2. Mixtures

In the previous section we have explored the effect of different parameters on the behavior of homopolymer melts. In this section, we examine the behavior of mixtures of liquid crystalline polymers characterized by different stiffness and/or molecular weight. To this end, we consider mixtures composed of polymers with the same segment length *b*, and the same orientational coupling *μ*. We also consider samples for which the Flory-Huggins parameter is zero and the segment volumes are identical. Thus, the only chemical feature is encoded into the chain stiffness parameters $\kappa_{\mathrm{stiff}}$ and $\kappa_{\mathrm{coil}}$, characterizing the stiff and flexible component, respectively. Let $f_{\mathrm{coil}}$ be the volume fraction of the flexible macromolecule in the mixture and $N_{\mathrm{coil}}$ the corresponding polymerization index. We consider the case where the stiffer macromolecule has a polymerization index $N_{\mathrm{stiff}}=16$. Simulations started from random initial configurations and evolved for 4 × $10^{6}$ MC steps. The simulation box size was kept fixed at L=64b to minimize finite-size effects. We consider the parameter space ($f_{\mathrm{coil}}$, $N_{\mathrm{coil}}$) for different systems characterized by several values of the orientational coupling. As before, the identification of single phase or phase separated morphologies was performed by visual inspection.

**Figure 5:**
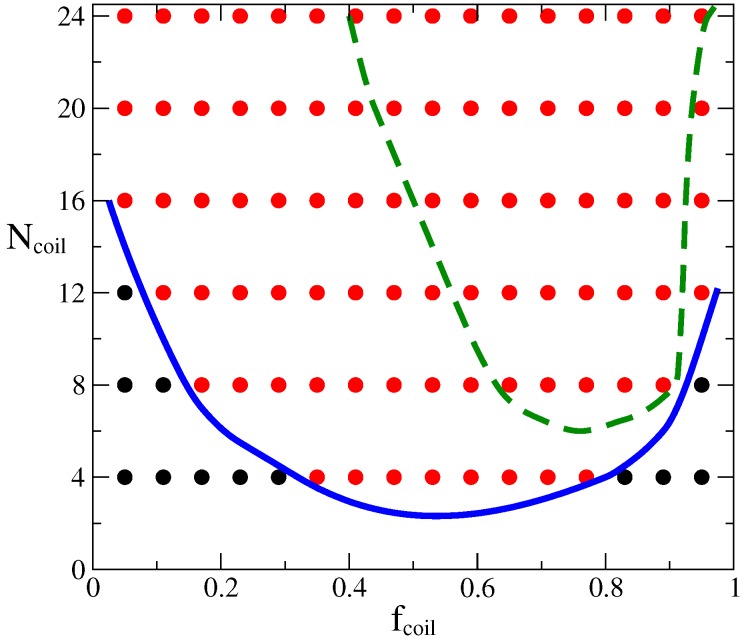
Phase diagram in the parameter space ($f_{\mathrm{coil}}$, $N_{\mathrm{coil}}$) for a mixture of rigid and flexible polymers, obtained by Monte Carlo simulations. Red circles indicate the points in the phase diagram that give rise to phase separated samples while black circles represent those conditions for which a single phase is obtained. Lines are only a guide to the eye and highlight the phase boundary, these lines were obtained by using splines on the simulation data. Symbols and continuous (blue) line are results for $\kappa_{\mathrm{coil}}=0.25$ ($\ell_{p}\approx 0.44b$), $\kappa_{\mathrm{stiff}}=5.0$ ($\ell_{p}\approx 4.5b$) and μ=4.0. The dashed green line is the phase boundary obtained for a sample with less dissimilarity between polymers, characterized by $\kappa_{\mathrm{coil}}=2.0$ ($\ell_{p}\approx 1.6b$), $\kappa_{\mathrm{stiff}}=5.0$ and μ=3.5.

Our first results were obtained by considering two highly dissimilar flexibilities with μ=4.0. For the latter value the stiff component, which is characterized by a stiffness parameter $\kappa_{\mathrm{stiff}}=5.0$, is deep into the nematic phase (see Figure 2). Thus, these macromolecules prefer to organize themselves into well aligned structures. However, the other component possesses a low stiffness parameter, we have chosen it to be $\kappa_{\mathrm{coil}}=0.25$. Under these conditions, the latter polymer melt is always in an isotropic phase, within the range of orientational strengths explored here. The chains are so flexible that configurational entropy overcomes the effect of the preferential alignment of polymer segments. When these two macromolecules with completely different flexibilities, but otherwise identical, are mixed together one could expect a strong dependence on the composition over the behavior of the system as a whole. It is found that the mixtures can be either in a single-phase where there is a homogeneous distribution of both kinds of polymers, or they can phase separate into domains, where nematic and isotropic phases coexist in the sample. In Figure 5 the phase behavior of this mixture in the parameter space ($f_{\mathrm{coil}}$, $N_{\mathrm{coil}}$) is presented. For these very dissimilar flexibilities, most of the conditions give rise to macroscopic phase separated morphologies (red circles). However, there are small regions where a single phase is present (black circles). For the latter case, one can distinguish two different single-phase states: for low volume fraction, $f_{\mathrm{coil}}$, and small molecular weight of the flexible component, the stiff macromolecules are aligned in a nematic phase and induce the alignment of the flexible chains, even though they would prefer to be in a disordered state in the bulk. This nematically-induced unfolding is viable if the molecular weight of the flexible macromolecules is not that large, such that the entropic cost of the unfolding of the coil molecules is compensated by the orientational interactions. However, as the polymerization index increases there, will be a value beyond which configurational entropy dominates over the nematic alignment, as it is obtained for $N_{\mathrm{coil}}\geq 16$ in the current system. On the other hand, for $f_{\mathrm{coil}}\to 1$, the great majority of coil chains induce coil configurations on the stiff polymers. Again, this disorder induced by disorder is the result of conformational entropy gains associated with the large numbers of small flexible chains. We should note here that, theoreticallly, a single phase can still exist for much larger $N_{\mathrm{coil}}$, with the width of the one-phase stability region, Δ→0 as the two extremes of the volume fraction are reached, $f_{\mathrm{coil}}\to 0,1$. Our modeling does not capture that, as we have considered a finite parameter set to explore the overall phase behavior.

**Figure 6:**
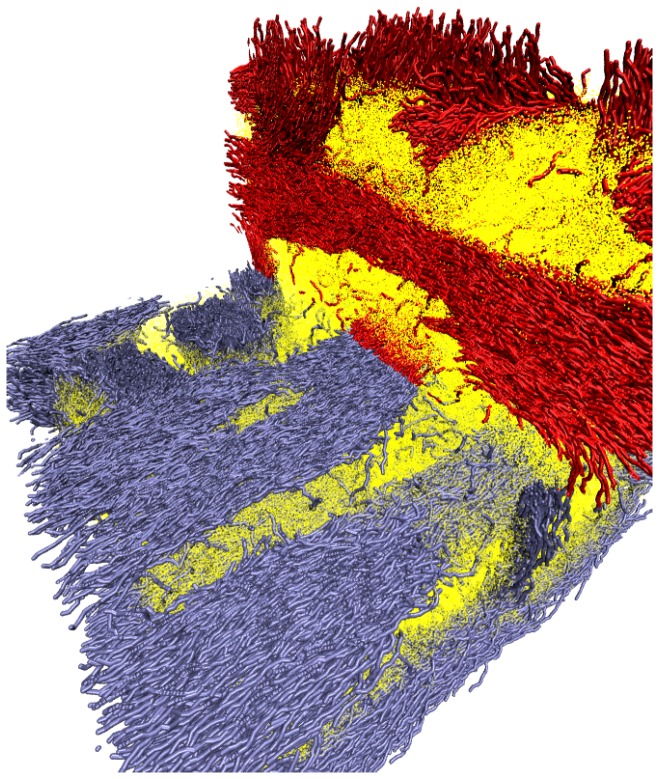
Three-dimensional cross-section of an instantaneous polymer configurations in the phase separated state, stiff polymer chains are shown in red and blue, segments of flexible chains are shown as yellow dots to facilitate visualization [45].

When macrophase separation occurs the system organizes into nematic and isotropic domains (this has been also observed experimentally, see for example Ref. [43]). As the initial configurations were homogeneous, and simulation box sizes are relatively large, we observe the local arrangement of stiff polymers into bundles with nematic order (nematic domains), without large-scale phase separation. The orientation of such nematic domains was, as expected, random. When the volume fraction of the stiff macromolecules was large enough, those nematic domains formed a percolating network through the whole sample (see Figure 6). To highlight this structure we have computed the local composition scalar field, ψ(r), defined by

(5) $$\psi \left(r\right)=\langle \frac{\phi_{\mathrm{stiff}}\left(r\right)-\phi_{\mathrm{coil}}\left(r\right)}{\phi_{\mathrm{stiff}}\left(r\right)+\phi_{\mathrm{coil}}\left(r\right)}\rangle .$$

**Figure 7:**
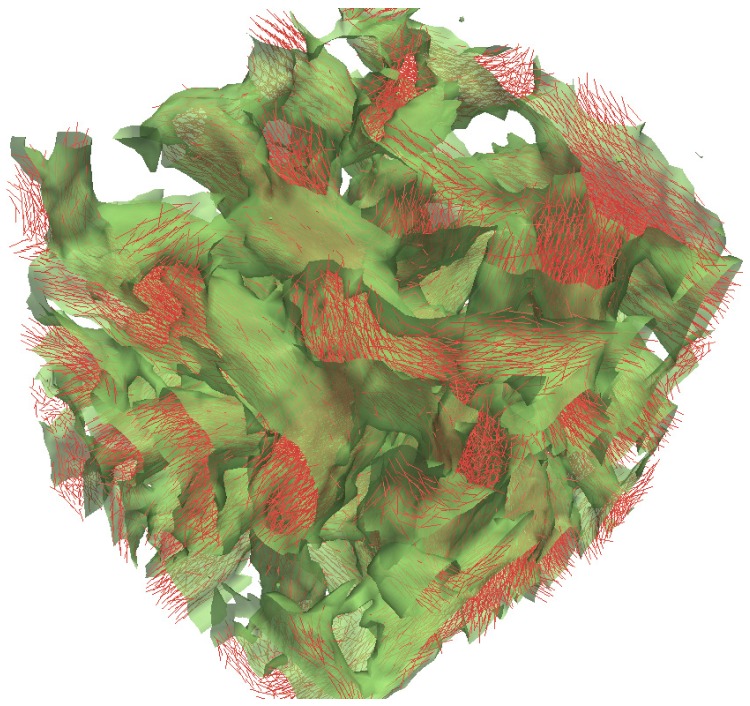
Iso-surface of the composition scalar parameter ψ(r)=0 corresponding to the interface between the rigid and flexible domains (green surface). Red lines are the local nematic director $\hat{n}\left(r\right)$ where local ordering S(r)≥0.7 [46].

The regions where ψ=−1,0 or +1 represent the coil-rich domains, the interface between the stiff and coil regions, and the stiff-rich domains, respectively. Additionally, we have measured the local orientational ordering by computing the local nematic director $\hat{n}\left(r\right)$ and the local scalar order parameter S(r) associated to the tensor order parameter Q(r). Figure 7 displays this combined information in a single plot. The interface between nematic and isotropic phases (ψ(r)=0) is shown as a green semi-transparent surface. For this example, $N_{\mathrm{coil}}=16$ and $f_{\mathrm{coil}}\approx 0.6$, thus the minority phase is composed by the stiff polymers and the interface bounds such material. As can be seen, such surface is formed by tube-like regions connected to each other in a percolating network. In the same plot, we have displayed the local nematic director (short red lines) where the local scalar order parameter S(r)≥0.7. As can be seen in the Figure 6, those regions of high orientational order correspond to points bounded by the interface between flexible and rigid macromolecules. This data also supports the fact that, locally, each tube-like region contains stiff polymers oriented in different directions. It should be noted here that such percolating structure is a non-equilibrium state; the true equilibrium should be one in which two macroscopic phases appear in the sample. However, because we use local and semi-local MC moves, the system gets trapped in this non-equilibrium state, as the stiff macromolecules form bundles oriented in a random direction, that represents, locally, a minimum energy state. Reaching equilibrium would require the alignment of all domains in a single uniform direction, which would take a long-time using the MC method adopted here. By decreasing the dissimilarity between the polymers in the mixture, the region of macrophase separation shrinks; this is shown by the phase boundary (green dashed line) displayed in Figure 5 for the case of stiff and flexible polymers characterized by $\kappa_{\mathrm{coil}}=2.0$, $\kappa_{\mathrm{stiff}}=5.0$ and μ=3.5. It is important to highlight that recent experimental results have shown that short-range intermolecular aggregation [17] and high persistence length [16] are critical to achieve efficient charge transport and high optical absorption, respectively. Thus, the results presented in this work can serve as useful as a guide for experimental formulation of these macromolecular materials.

We noted here that in this work we have used the simplest model, where $\mu_{\mathrm{coil}}=\mu_{\mathrm{stiff}}=\mu$, as its implementation is straightforward and provides physical insights into the molecular level organization that occurs in these polymer blends. The generalization to dissimilar orientational couplings is straightforward, and one would expect that in the particular case with $\mu_{\mathrm{coil}}\ll \mu_{\mathrm{stiff}}$, the region of macrophase separation will be wider (Figure 4). This more realistic case is important, in particular, for the case of block polymers self-assembly [44]; we are currently pursuing research on this topic.

## 4. Conclusions

In this work we have used a coarse-grained formalism to study the phase behavior of semi-flexible nematic polymers in pure melts and mixtures. We have explored the conditions that lead to macrophase separation in mixtures of stiff and flexible polymers mediated by the orientational interaction in otherwise identical polymers. We have found that in macroscopic samples the kinetics of phase separation will lead to formation of non-equilibrium morphologies where nematic domains form a percolating network that spans the whole sample. The latter structures are of interest for the development of photovoltaic organic devices. The formalism used in this work should therefore provide a strategy for systematic coarsening from atomistic simulations to coarse-grained representations and vice versa.
